# Outbreak of *Burkholderia stabilis* Infections Associated with Contaminated Nonsterile, Multiuse Ultrasound Gel — 10 States, May–September 2021

**DOI:** 10.15585/mmwr.mm7148a3

**Published:** 2022-12-02

**Authors:** Matthew J. Hudson, Stacy C. Park, Amy Mathers, Hardik Parikh, Janet Glowicz, David Dar, Marjan Nabili, John J. LiPuma, Amy Bumford, Matthew A. Pettengill, Mark R. Sterner, Julie Paoline, Stacy Tressler, Tiina Peritz, Jane Gould, Stuart R. Hutter, Heather Moulton-Meissner, Kiran M. Perkins

**Affiliations:** ^1^Epidemic Intelligence Service, CDC; ^2^School of Medicine, University of Virginia, Charlottesville, Virginia; ^3^Division of Healthcare Quality Promotion, National Center for Emerging and Zoonotic Infectious Diseases, CDC; ^4^Food and Drug Administration, Silver Spring, Maryland; ^5^University of Michigan Medical School, Ann Arbor, Michigan; ^6^Sidney Kimmel College of Medicine, Thomas Jefferson University, Philadelphia, Pennsylvania; ^7^Pennsylvania Department of Health; ^8^Philadelphia Department of Public Health, Philadelphia, Pennsylvania; ^9^Virginia Department of Health.

In July 2021, the Virginia Department of Health notified CDC of a cluster of eight invasive infections with *Burkholderia stabilis*, a bacterium in the *Burkholderia cepacia* complex (BCC), among hospitalized patients at hospital A. Most patients had undergone ultrasound-guided procedures during their admission. Culture of MediChoice M500812 nonsterile ultrasound gel used in hospital A revealed contamination of unopened product with *B. stabilis* that matched the whole genome sequencing (WGS) of *B. stabilis* strains found among patients. CDC and hospital A, in collaboration with partner health care facilities, state and local health departments, and the Food and Drug Administration (FDA), identified 119 *B. stabilis* infections in 10 U.S. states, leading to the national recall of all ultrasound gel products produced by Eco-Med Pharmaceutical (Eco-Med), the manufacturer of MediChoice M500812. Additional investigation of health care facility practices revealed frequent use of nonsterile ultrasound gel to assist with visualization in preparation for or during invasive, percutaneous procedures (e.g., intravenous catheter insertion). This practice could have allowed introduction of contaminated ultrasound gel into sterile body sites when gel and associated viable bacteria were not completely removed from skin, leading to invasive infections. This outbreak highlights the importance of appropriate use of ultrasound gel within health care settings to help prevent patient infections, including the use of only sterile, single-use ultrasound gel for ultrasonography when subsequent percutaneous procedures might be performed.

## Investigation and Results

On July 21, 2021, the Virginia Department of Health notified CDC that eight patients with invasive *B. stabilis* infection (mostly bloodstream infections) had been identified by hospital A during May 18–July 20, 2021. At least seven of the eight patients had undergone ultrasound-guided procedures at hospital A. Unopened bottles of nonsterile ultrasound gel, MediChoice M500812, present at the facility were sampled and cultured. Initial cultures identified BCC organisms in eight of 13 unopened bottles; subsequent WGS identified BCC as *B. stabilis* among bottles representing three lots of MediChoice M500812 ultrasound gel. Quantitative testing yielded high bacterial bioburden (7.0 x 10^6^–5.8 x 10^7^ colony-forming units/mL) in bottles from two of these lots. The genetic sequences of *B. stabilis* for all eight clinical (seven from blood and one from ascites fluid) and three product isolates collected at hospital A were closely related (0–11 single nucleotide variants with coverage of >99% of the full reference genome). Hospital A reported these results to CDC on July 23, 2021.

During the week of July 18, 2021, hospital A posted a query regarding unusual BCC blood cultures on an American Society of Microbiology Listserv. On July 22, the Philadelphia Department of Public Health notified CDC about seven patients in an acute care hospital (hospital B) with BCC bloodstream infections identified during July 7–July 20, 2021, four of whom had undergone ultrasound-guided percutaneous procedures. Hospital B cultured bottles from 21 lots of ultrasound gel and identified BCC in two of these lots, including one of the three lots in which BCC had previously been identified by hospital A and an additional fourth lot of unopened MediChoice M500812 ultrasound gel. Hospital B shared these clinical and product isolates with hospital A for WGS, which confirmed isolates to be *B. stabilis* and demonstrated that patient and product isolates from the two facilities were closely related (1–7 single nucleotide variants, >99% genome coverage), raising concern about contamination of the ultrasound gel during manufacturing or distribution. Although nonsterile, multiuse ultrasound gel is intended only for external, noninvasive ultrasonography (e.g., transthoracic echocardiogram and diagnostic abdominal ultrasound), both hospitals noted that health care personnel often use this ultrasound gel to visualize anatomic structures during percutaneous procedures (e.g., locating veins to guide peripheral intravenous catheter insertion). This practice could have left gel containing viable bacteria on the skin that is difficult to remove before the procedure, preventing adequate skin antisepsis and allowing introduction of BCC into sterile body sites.

CDC subsequently collected information on demographic and clinical characteristics for any patients with *B. stabilis* infections reported to CDC during July 21–October 15, 2021, with the assistance of state and local health departments, which collected this information from health care facilities. CDC also facilitated sharing of isolates and WGS information among facilities with patient infections and hospital A, which conducted WGS comparisons for isolates among facilities reporting cases. The University of Michigan *Burkholderia cepacia* Research Laboratory and Repository performed repetitive extragenic palindromic polymerase chain reaction (rep-PCR) for selected isolates. For this investigation, a case was defined as a positive culture for *B. stabilis* in a patient specimen collected from any body site on or after January 1, 2021, in which the isolate was genetically related to the outbreak strain by WGS (match within 12 single nucleotide variants, >99% coverage across the entire *B. stabilis* reference genome) or rep-PCR (match defined as similarity coefficient >85%).

CDC was notified of 119 *B. stabilis* patient infections among 10 states meeting the case definition (Table). Reported isolates were collected during May 15–September 14, 2021. The median patient age was 61 years (range = 4 days–92 years). Median interval from hospital admission to detection of *B. stabilis* infection was 1 day (range = 0–118 days). Most infections were bloodstream infections (106, 89%). Among 87 patients with available clinical data, 59 (68%) had signs and symptoms of infection (e.g., fever and tachycardia). Among 102 patients with vital status information, 14 (14%) deaths were reported during the hospitalization in which *B. stabilis* infection was identified. Cause of death was available for 10 patients and was attributed to *B. stabilis* infection in two of these. Cause of death for the remaining eight patients included septic shock unrelated to BCC (three), cardiac arrest (two), hypoxemic respiratory failure (one), respiratory failure secondary to COVID-19 (one), and sickle cell crisis (one). Among 117 patients with available information, 104 (89%) are known to have undergone ultrasonography during their admission, and 103 (94%) underwent an ultrasound-associated percutaneous procedure (e.g., peripheral intravenous catheter insertion or paracentesis). An Eco-Gel 200 product was documented to have been used among 31 (26%) of all infections and was known to have been present in all facilities reporting cases.

**TABLE:** Demographics, clinical characteristics, and exposures of patients with *Burkholderia stabilis* infections associated with contaminated ultrasound gel (N = 119) — United States, May–September 2021

Characteristic (no. with available information)	No. (%)
**Age, yrs, median (range) (n = 68)**	61 (4 days–92 yrs)
**Sex (n = 89)**	
Female	44 (49)
Male	45 (51)
**Jurisdiction (n = 119)**	
California	12 (10)
Illinois	6 (5)
Minnesota	23 (19)
New Jersey	4 (3)
New Mexico	1 (1)
New York	6 (5)
Ohio	4 (3)
Pennsylvania (not including Philadelphia)	19 (16)
Philadelphia	35 (29)
Virginia	8 (7)
Washington	1 (1)
Signs and symptoms of infection* (n = 87)	59 (68)
**Site of infection (n = 119)**	
Blood	106 (89)
Ascites or abdominal fluid	5 (4)
Sputum	3 (3)
Wound	3 (3)
Amniotic fluid	1 (1)
Bile	1 (1)
**Days from admission to detection of infection, median (range) (n = 113)**	1 (0–118)
**Treated for *Burkholderia cepacia* complex infection (n = 63)**	51 (81)
**Deaths (n = 102)^†^**	14 (14)
**Underwent ultrasonography during admission (n = 117)**	104 (89)
Number of ultrasounds during admission, mean (range)	1.8 (0–11)
**Underwent ultrasound-guided percutaneous procedure (n = 109)**	103 (94)
Peripheral intravenous catheter placement	59 (57)
Central venous catheter (includes peripherally inserted central catheter and hemodialysis catheter)	14 (14)
Arterial line	10 (10)
Paracentesis	7 (7)
Aspiration of fluid collection	4 (4)
Thoracentesis or chest tube	3 (3)
Nerve block	2 (2)
Percutaneous biopsy of lesion	2 (2)
Amniocentesis	1 (1)
Gallbladder aspiration	1 (1)
**Underwent intracavitary ultrasound^§^ (n = 100)**	3 (3)
**Hospital location where ultrasound was performed (n = 72)^¶^**	
Emergency department or trauma bay	34 (47)
Inpatient room	24 (33)
Radiology suite	9 (13)
Operating room	6 (8)
Outpatient clinic	5 (7)
**Known exposure to Eco-Med 200 product (n = 39)**	31 (79)

* Signs and symptoms of infection included fever, tachycardia, and leukocytosis. It is hypothesized that a proportion of blood cultures were positive for *Burkholderia cepacia* complex without sign of infection because of specimen contamination, whereby the specimen was drawn directly at the site where the ultrasound gel had been applied and not completely removed.

^†^ Cause of death was only available for 10 patients and was attributed to *Burkholderia* *stabilis* infection in two of these. Cause of death for the remaining eight patients included septic shock unrelated to *Burkholderia cepacia* complex (three), cardiac arrest (two), hypoxemic respiratory failure (one), respiratory failure secondary to COVID-19 (one), and sickle cell crisis (one).

^§^ All intracavitary ultrasound procedures were transesophageal echocardiograms.

^¶^ Categories are not mutually exclusive.

## Public Health Response

Because of the concern for product contamination, CDC notified FDA on July 23, 2021, of the epidemiologic and laboratory findings. FDA and CDC informed Eco-Med on July 29, 2021, of the patient infections, resulting in a voluntary recall of eight product lots on August 4, 2021, including the four lots initially identified by hospitals A and B (*1*). The recall also advised facilities to quarantine all associated products from Eco-Med, including all MediChoice M500812 gel and its other ultrasound gel product line, Eco-Gel 200, while investigation was ongoing (*1*). On August 4, 2021, CDC issued an Epidemic Information Exchange communication to relevant professional organizations to alert public health and clinical communities of the infections and product recall (*2*).

Additional FDA investigation of manufacturing protocols revealed concern for potential bacterial product contamination beyond the eight recalled lots, in light of the company’s inappropriate testing of finished product, inadequate testing of raw materials, and a lack of environmental controls, although the root cause and extent of the bacterial contamination was not identified (*3*). On August 18, 2021, FDA advised immediate discontinuation of use and discarding of all ultrasound gels and lotions manufactured by Eco-Med (*3*). The manufacturer ceased operation and FDA engaged the multiple distributors of the product to ensure execution of an expanded recall of all ultrasound gels and lotions manufactured by Eco-Med. After the recall, FDA also collected samples of product from distributor sites and a point of importation for laboratory analysis and confirmation of contamination. Subsequent FDA testing identified bacterial contamination in eight of the 13 tested lots of ultrasound gel manufactured by Eco-Med, seven of which were contaminated with BCC (and an additional lot contaminated with *Bacillus circulans*). One of these contaminated lots had been identified by hospital A; the other seven were additional lots not included in the original product recall, validating FDA’s recommendation for expansion of the initial recall.

Health departments in cities and states with facilities reporting cases reported that all affected facilities removed all ultrasound gels and lotions manufactured by Eco-Med from clinical areas and destroyed the products or returned them to their distributors. No additional cases were reported to CDC after October 12, 2021.

## Discussion

BCC is a group of opportunistic pathogens with intrinsic resistance to certain preservatives and antimicrobial agents often used in aqueous products and can cause clinical infection in healthy persons who are exposed to contaminated medical products or devices (*4*,*5*). Infection with BCC has been associated with ultrasound gel in previous outbreaks (*4*–*6*). In this outbreak, BCC-contaminated ultrasound gel was likely introduced into sterile body sites during invasive procedures when needles were advanced through skin on which the contaminated gel had been applied before or during the procedure. Such practices, including the routine use of ultrasonography and multiuse ultrasound gel to guide peripheral intravenous catheter placement, were reported as occurring in affected facilities across multiple jurisdictions. Only single-use, sterile ultrasound gel packets should be used for ultrasonography in anticipation of, preparation for, or during percutaneous procedures (*7*). Ultrasound probes and other related devices (e.g., consoles and handles) should also be completely cleaned and disinfected according to manufacturers’ instructions to avoid the transmission of pathogens to patients (*7*). A high bioburden of bacteria noted on quantitative testing and BCC’s intrinsic resistance to antiseptics commonly used in clinical practice might have further contributed to this outbreak by rendering skin antiseptics less effective when used as part of aseptic preparation for such procedures (*8*,*9*). After all external ultrasonographic examinations, ultrasound gel should be thoroughly removed from the skin, and care must be taken to ensure that any residual gel is completely cleaned off. Once all residual ultrasound gel is removed, skin antisepsis as indicated for the procedure should be performed at the site before proceeding with any associated invasive procedure. Additional considerations for the appropriate use of ultrasound gel might also prevent infections (Box).

Box Considerations for the use of ultrasound gel*

**Sterile ultrasound gel**

- Use single-use, sterile ultrasound gel for ultrasonography performed in preparation for or during percutaneous procedures (e.g., placement of central and peripheral intravenous lines, amniocentesis, paracentesis, tissue biopsy, and surgical procedures).^†^
  - Do not use nonsterile ultrasound gel for visualization before such procedures.
  - If nonsterile ultrasound gel is inadvertently used before such procedures (e.g., unanticipated procedure), care must be taken to ensure that all residual gel is removed from the skin and the appropriate skin antisepsis is performed before the procedure.
- Use single-use, sterile ultrasound gel for all ultrasound procedures performed on nonintact skin or near fresh surgical sites.^†^
- Whenever feasible, use single-use, sterile ultrasound gel inside single-use or sterile ultrasound probe covers.^†^

**Nonsterile ultrasound gel**

- If multiuse containers are used^†^:
  - Do not refill; discard and replace multidose containers when empty.
  - Seal container when not in use.
  - Avoid direct contact between gel container dispensing tip and any persons or instrumentation, including the ultrasound transducer.
- If a patient under contact precautions undergoes an ultrasound using gel dispensed from a multiuse container, discard the container after use.^†^
- After ultrasonography, clean the skin, ensuring that all residual ultrasound gel is removed.^§^

**Reprocessing of ultrasound equipment**

- Follow manufacturer’s instructions for ultrasound probe reprocessing to ensure recommended cleaning and disinfection protocols are being followed.^†^^,^^¶^
- Clean and thoroughly disinfect ultrasound consoles and other parts of the ultrasound device that do not come into direct contact with the patient (e.g., handles, cables, connectors, and holders) and any warming devices or other noncritical surfaces associated with ultrasound procedures before use on another patient.^†^ Containers for ultrasound gel and consoles should be considered high-touch surfaces.
- All transducers used on either mucous membranes or nonintact skin (e.g., use in transvaginal, transrectal, and transesophageal procedures) require high-level disinfection or sterilization before use on another patient.^†^^,^^§^^,^^¶^^,^**

* For all ultrasonography, standard precautions including adherence to hand hygiene and the use of personal protective equipment are recommended. Surgical hand scrub and use of sterile barriers is recommended for sterile procedures.

^†^ https://www.aium.org/officialstatements/57

^§^ https://www.cdc.gov/infectioncontrol/guidelines/disinfection/

^¶^ https://doi.org/10.1002/jum.15653

** https://www.fda.gov/media/71100/download

This investigation highlights that BCC can pose a risk for invasive infections because of contamination of nonsterile aqueous medical products even when intended use is limited to skin. Other, nonsterile aqueous medical products implicated in health care–associated outbreaks due to BCC contamination include nasal sprays, mouthwashes, preoperative skin solutions, and hand sanitizers, among others. Manufacturers of water-based medical products and medical devices (e.g., ultrasound gels) should ensure that quality system processes include pathogen prevention and identification as part of their contamination and environmental control requirements.^†^ Health care personnel should be trained for the appropriate use of ultrasound gel associated with ultrasounds and ultrasound-associated procedures, including that only sterile, single-use ultrasound gel should be used before and during invasive percutaneous procedures to prevent additional outbreaks of serious patient infections (*7*).

**Summary**

**What is already known about this topic?**

*Burkholderia cepacia* complex (BCC) is a group of opportunistic pathogens that can cause infection in healthy persons who become exposed to contaminated medical products.

**What is added by this report?**

In 2021, a total of 119 BCC infections were associated with multiple lots of nonsterile ultrasound gel contaminated with BCC organisms. Use of this contaminated gel before percutaneous procedures likely contributed to patient infections.

**What are the implications for public health practice?**

Ensuring quality system practices during manufacturing and appropriate use of products in clinical practice are crucial to preventing infections. Health care personnel who perform ultrasounds and ultrasound-associated procedures should be trained for the appropriate use of ultrasound gel associated with these procedures.

## Footnotes

* These authors contributed equally to this report.

† Quality System Regulation, 21 C.F.R. 820.70 (2022). Items c and e. https://www.accessdata.fda.gov/scripts/cdrh/cfdocs/cfcfr/CFRSearch.cfm?fr=820.70
